# Isolation of Mouse Cerebral Microvasculature for Molecular and Single-Cell Analysis

**DOI:** 10.3389/fncel.2020.00084

**Published:** 2020-04-09

**Authors:** Hallel C. Paraiso, Xueqian Wang, Ping-Chang Kuo, Destin Furnas, Barbara A. Scofield, Fen-Lei Chang, Jui-Hung Yen, I-Chen Yu

**Affiliations:** ^1^Department of Anatomy, Cell Biology, and Physiology, Indiana University School of Medicine, Fort Wayne, IN, United States; ^2^Department of Pharmacology and Toxicology, Indiana University School of Medicine, Fort Wayne, IN, United States; ^3^Department of Microbiology and Immunology, Indiana University School of Medicine, Fort Wayne, IN, United States; ^4^Department of Neurology, Indiana University School of Medicine, Fort Wayne, IN, United States

**Keywords:** blood-brain barrier, endothelial cells, brain capillaries, single-cell isolation, microvasculature damage

## Abstract

Brain microvasculature forms a specialized structure, the blood-brain barrier (BBB), to maintain homeostasis and integrity of the central nervous system (CNS). The BBB dysfunction is emerging as a critical contributor to multiple neurological disorders, including stroke, traumatic brain injury, autoimmune multiple sclerosis, and neurodegenerative diseases. The brain microvasculature exhibits highly cellular and regional heterogeneity to accommodate dynamic changes of microenvironment during homeostasis and diseases. Thus, investigating the underlying mechanisms that contribute to molecular or cellular changes of the BBB is a significant challenge. Here, we describe an optimized protocol to purify microvessels from the mouse cerebral cortex using mechanical homogenization and density-gradient centrifugation, while maintaining the structural integrity and functional activity of the BBB. We show that the isolated microvessel fragments consist of BBB cell populations, including endothelial cells, astrocyte end-feet, pericytes, as well as tight junction proteins that seal endothelial cells. Furthermore, we describe the procedures to generate single-cell suspensions from isolated microvessel fragments. We demonstrate that cells in the single-cell suspensions are highly viable and suitable for single-cell RNA-sequencing analysis. This protocol does not require transgenic mice and cell sorting equipment to isolate fluorescence-labeled endothelial cells. The optimized procedures can be applied to different disease models to generate viable cells for single-cell analysis to uncover transcriptional or epigenetic landscapes of BBB component cells.

## Introduction

The brain vasculature is a complex system composed of endothelial cells, pericytes, astrocytes, smooth muscle cells, and extracellular matrix components, forming the unique blood-brain barrier (BBB), which lies at the interface between circulating blood and the neural tissue (Abbott and Friedman, 2012; Obermeier et al., 2013). The BBB tightly regulates the transport of ions and nutrients necessary for neuronal health and function. It also prevents harmful substances or cells from entering into the central nervous system (CNS). Previous literature suggests that the BBB disruption can lead to neuronal injury mediated through peripheral immune cells or harmful agents that enter into the CNS in multiple neurological disorders, such as Alzheimer’s disease (AD; Sweeney et al., 2018; Nation et al., 2019), ischemic stroke (Kassner and Merali, 2015), multiple sclerosis (Ortiz et al., 2014), and traumatic brain injury (Alluri et al., 2015). Despite the importance of BBB highlighted in many neurological disorders, the molecular and cellular mechanisms that lead to BBB dysfunction or disruption in these disease conditions remain largely unknown. Therefore, it is vital to develop a method to isolate brain microvasculature with preserved structural integrity to identify subtle aberrations of the BBB in disease animal models. Here, we describe an optimized protocol that would allow isolating brain microvessels that closely resemble *in vivo* structures suitable for downstream applications of molecular and single-cell analyses to characterize molecular signatures of BBB component cells.

## Materials and Equipments

### Animals

C57BL/6 and *db/db* mice were purchased from the Jackson Laboratory and bred at the animal facility of Indiana University School of Medicine. Mice were housed and maintained at 25°C under a 12 h light/ 12 h dark cycle with ad libitum access to food and water. Adult female mice aged 12–16 weeks or 9 months were used for the present study. All animal procedures in this study were conducted following the National Institutes of Health (NIH) Guide for the Care and Use of Laboratory Animals and approved by Purdue Animal Care and Use Committee.

### Reagents

(1) Sigma–Aldrich Potassium Chloride (SKU: P9541-500G)
(2) Baker Analyzed^®^ Potassium Phosphate Monobasic, Crystal (CAS: 7778-77-0)
(3) Fisher Sodium Chloride (CAS: 7647-14-5)
(4) Fisher Sodium Phosphate Dibasic Anhydrous (CAS: 7558-79-4)
(5) Fisher Calcium Chloride Dihydrate (CAS: 10035-04-8)
(6) Fisher Magnesium Chloride (CAS: 7791-18-6)
(7) Sigma Aldrich D-(+)-glucose (SKU: G8270-100G)
(8) Sigma–Aldrich Sodium Pyruvate (Product number: P2256)
(9) Thermo Fisher Scientific GE Healthcare Ficoll PM400 (Catalog number: 45-001-745)
(10) Elko Filtering Co 30 micron nylon mesh (Catalog number: NC0478162)
(11) Sefar, 03-100/32, Nylon Mesh Filtering Screen 100 Micron—Open Area %: 32— Width: 38 in, Natural Color (1 Yard; Part number: 3A03-0100-098-00)
(12) Alkali Scientific Inc. 100 micron strainer (Catalog number: TS100)
(13) Thermo Fisher Scientific Sartorius™ glass beads (0.4 mm–0.6 mm; Catalog number: BBI-8541701)
(14) Sigma–Aldrich Bovine Serum Albumin (BSA; Catalog number: A2153)
(15) Sigma–Aldrich Collagenase (Catalog number: C5138)
(16) Sigma–Aldrich DNase I (Catalog number: DN25)
(17) BioLegend APC/Cy7 anti-mouse CD45 (clone: 30-F11)
(18) BioLegend Alexa Fluor 488 anti-mouse CD31 (clone: MEC13.3)
(19) BioLegend 7-AAD Viability Staining
(20) Miltenyi Biotec APC anti-mouse ACSA2 (clone: IH3-18A3)
(21) Miltenyi Biotec FcR blocking reagent—mouse
(22) BioLegend anti-mouse CD140b (clone: APB5)
(23) BD Bioscience anti-mouse CD31 (clone: MEC13.3)
(24) Sigma–Aldrich Rabbit anti-Aqp4 (Catalog number: HPA014784)
(25) Thermo Fisher Scientific Rabbit anti-ZO-1 (Catalog number: 61-7300)
(26) Proteintech Rabbit anti-Occludin (Catalog number: 13409-1-AP)
(27) BD Bioscience Mouse anti-actin (Catalog number: 612656)
(28) Sigma–Aldrich Phosphate Buffered Saline with 10% Bovine Albumin (BSA; Catalog number: SRE0036)
(29) Anaspec HiLyte™ Fluor 488 labeled human amyloid beta-peptide 1–42 (Catalog number: AS-60479-01)
(30) Sigma–Aldrich PSC833 (Catalog number: SML0572)

### Isolation Equipment

(1) Milwaukee $\frac{1}{2}$ in (13 mm) drill (Catalog number: 0299-20)
(2) Wheaton Dounce homogenizer—7 ml (Catalog number: 3432T40)
(3) Staco Energy Products Variable Autotransformer (Model: 3PN1010B)
(4) Beckman Coulter Avanti J-E centrifuge
(5) Beckman JA-20 rotor (20,000 rpm)
(6) Fotodyne Stovall The Belly Dancer Hybridization Water Bath (SKU: 7121211)
(7) Eppendorf 5810 R Centrifuge

### Analysis Equipment and Software

(1) BD FACSVerse™
(2) Olympus FV10i confocal microscope
(3) Olympus DP72 light microscope
(4) Applied Biosystems StepOne Plus real-time PCR system
(5) NIH ImageJ software
(6) Prism 8

## Step-By-Step Procedures

### Day Before Experiment

(1) Prepare modified phosphate-buffered saline (PBS, 2.7 mmol/L KCl, 1.5 mmol/L KH_2_PO_4_, 136.8 mmol/L NaCl, 4.3 mmol/L Na_2_HPO_4_, 0.7 mmol/L CaCl_2_, 0.5 mmol/L MgCl_2_, pH 7.4). The volume of 500 ml modified PBS would be used for 3–5 mice.
(2) Prepare 8 ml of 40% Ficoll solution in modified PBS per group.
(3) Store both the modified PBS and the 40% Ficoll in 4°C.

### Day of Experiment

(1) Add D-glucose (5 mmol/L) and sodium pyruvate (1 mmol/L) to the modified PBS.
(2) Prepare 1% BSA in modified PBS.
(3) Place all buffers on ice and pre-cool all tools and centrifuges.

### Brain Microvessel Fragments for Molecular Assays

(1) Anesthetize mice using isoflurane. When the animals are asleep, sterilize the animals with 70% ethanol, and decapitate the animals. Extract brains as described in Figure 2 and transfer brains to a Petri dish containing ice-cold modified PBS. Carefully remove the thin slices of subdural meninges (SDM) on the dorsal surface of the brain. Use the dissecting microscope to validate the removal of SDM quickly. Keep the brain on ice.
(2) Using the brain matrix, remove olfactory bulbs and cerebellum. Cut the brain in half horizontally. Using Needle blade, separate the cortex from the corpus callosum. Place cortices in a glass tube containing 7 ml ice-cold modified PBS.
(3) In a 4°C cold room, homogenize cortices for 35 strokes using an electrical drill (Milwaukee drill connected to the Staco Energy Products Autotransformer)
  (a) Speed for the first 20 strokes: 18
  (b) Speed for the last 15 strokes: 20
  (c) Transfer homogenates to Dounce homogenizer and homogenize manually for additional 10 strokes.
(4) Mix brain homogenate with an equal volume of 40% Ficoll solution to a final concentration of 20% Ficoll PM 400. Shake tubes before centrifuge.
(5) The homogenate will be spun at 5,800× *g*, 4°C for 20 min.
(6) Discard supernatant and vacuum the myelin layer at the top.
(7) The pellet containing enriched microvessels can then be used for molecular assays such as RNA extraction or Western blot analysis. Add the appropriate volume of Trizol or RIPA buffer to the pellet and re-suspend it gently for downstream analyses.

**Figure 1:**
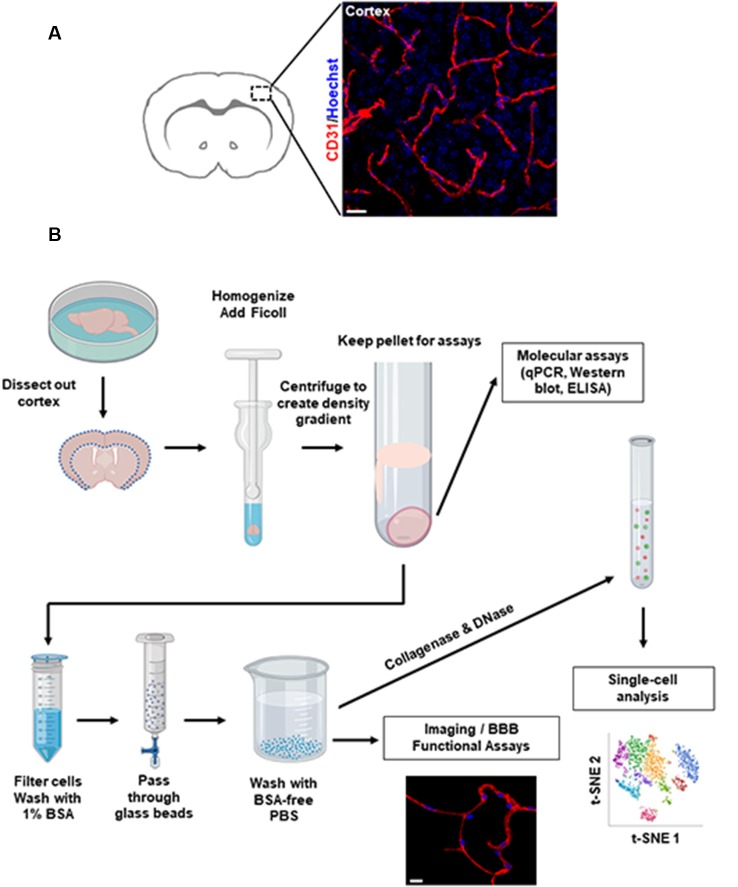
Overview of brain microvessel purification protocol. **(A)** Representative images of microvessels in the mouse cerebral cortex labeled by immunofluorescence staining. Scale bar, 20 μm. **(B)** Flowchart of the isolation procedures. This diagram depicts the major steps to isolate microvessels from freshly harvested mouse brain. Scale bar, 10 μm. Representative single-cell t-SNE plot shows different colored clusters to identify different cell types.

**Figure 2:**
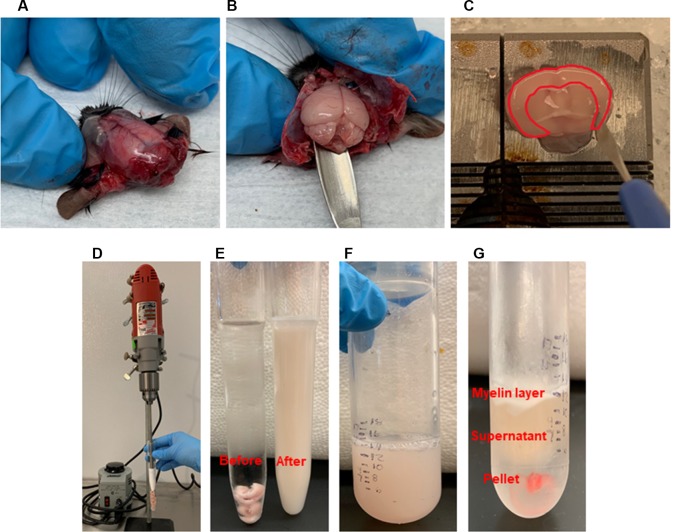
Brain microvessel enrichment. **(A–C)** Tissue harvest and dissection. After head decapitation, peeling the skin to reveal the skull. Extracting the brain with a spatula. After transferring the whole brain into a Petri dish containing modified phosphate buffered saline (PBS) on ice, dissecting the brain regions on ice. Dissecting the cerebral cortex was shown. Highlighted area showed the cortex which is cut out using a needle blade. **(D–G)**. Homogenization and Ficoll gradient enrichment. **(D)** Multi-speed drill homogenizes collected cortices. **(E)** Before and after homogenization. **(F)** An equal amount of 40% Ficoll is added to the homogenates and transferred to a centrifuge tube. **(G)** Post-centrifugation shows the myelin layer at the top and the pellet at the bottom, which contains the brain microvessels.

### Purification of Brain Capillaries for Imaging/Functional Assays

(1) In a 4°C cold room, take the pellet containing enriched microvessels from the previous step and re-suspend it into 1 ml of 1% BSA/modified PBS.
(2) Filter the solution with 300 μm nylon mesh and wash the mesh with 10 ml of 1% BSA/modified PBS and save the flow-through.
(3) Filter the flow through with 30 μm strainer very slowly by using a 7 ml transfer pipet. The speed of the droplet is 1 drop every 2 s. Capillaries will be captured on top of the strainer.
(4) Carefully invert the 30 μm strainer and wash with 10 ml of 1% BSA/modified PBS.
(5) Prepare an enrichment column in a 4°C room and add a volume of 15 ml glass beads to a 30 ml syringe supported by a 30 μm nylon mesh.
(6) Rinse the column with 10 ml of 1% BSA/modified PBS before adding the capillary solution.
(7) Transfer the capillary solution into the syringe by using a 7 ml transfer pipet.
(8) Gently agitate the beads, but do not touch the bottom 5 ml of beads.
(9) Adjust the flow rate so that the speed of the droplet is 1 drop per every 2 s.
(10) Rinse with 10 ml of 1% BSA/modified PBS one time, gently agitating the beads after adding the buffer.
(11) Rinse with 10 ml of BSA-free PBS twice, gently agitating the beads after adding the buffer.
(12) Brain capillaries will be bound by the glass beads. Collect capillaries by pouring the beads into a sterile glass beaker.
(13) Add 50 ml of BSA-free modified PBS and gently agitate by aspirating the beads with a 25 ml serological pipet.
(14) Filter the solution into a clean 50 ml Falcon tube supported by a 100 μm strainer to capture the glass beads.
(15) Repeat the wash step of glass beads one time with an additional 50 ml of BSA-free modified PBS and filter the solution into another clean 50 ml Falcon tube.
(16) Centrifuge 50 ml Falcon tubes at 1,100 rpm, 4°C for 5 min. At the end of the centrifugation, remove supernatant and combine the pellets into one tube.
(17) Wash the pellet with 50 ml BSA-free modified PBS for an additional two times.
(18) Re-suspend the pellet in 250 μl 1% BSA/modified PBS. The enriched capillaries can be examined by light microscopy.
(19) For imaging, load 20 μl of re-suspended capillaries on a glass coverslip, spreading in a Z-shaped manner gently.
(20) Let the capillaries attach to the glass coverslip at room temperature for 30 min.
(21) Wash with 1 ml of 1% BSA/modified PBS. After wash, the capillaries can be fixed with 2% paraformaldehyde and subjected to immunostaining analyses.

### Generation of Single-Cell Suspensions of Brain Microvessels

(1) After the glass bead purification step, the capillary pellet can be used to generate single-cell suspensions. Re-suspend the enriched capillary pellet in 2 ml of 1× HBSS^−/−^ buffer and transfer to a new 50 ml Falcon tube.
(2) Add 0.5 mg/ml collagenase, and incubate the solution in a water bath set to 37°C with gentle shaking for 15 min. Swirl the tube gently every 5 min.
(3) At the end of the incubation period, add 2 μg/ml DNase, and gently pipet up and down with a 1 ml wide-bore tip until aggregate is dissociated.
(4) Incubate the mixture in the hybridization water bath at 37°C for 5 min.
(5) At the end of the incubation period, immediately add 13 ml pre-warmed RPMI-164 media with 10% fetal bovine serum to quench the enzyme activity.
(6) Pass a total of 15 ml of the mixture through a 70 μm strainer and transfer to a new 50 ml Falcon tube. Centrifuge mixture at 1,100 rpm, 4°C for 5 min.
(7) Remove the supernatant carefully using a pipette and re-suspend the cell pellet with ice-cold buffer. Pass cell suspensions through a 40 μm FLOWMI strainer.
(8) The viability and cell yield can be examined. At this point, the cells are ready for downstream applications, such as single-cell analyses.

## Results

**Figure 3:**
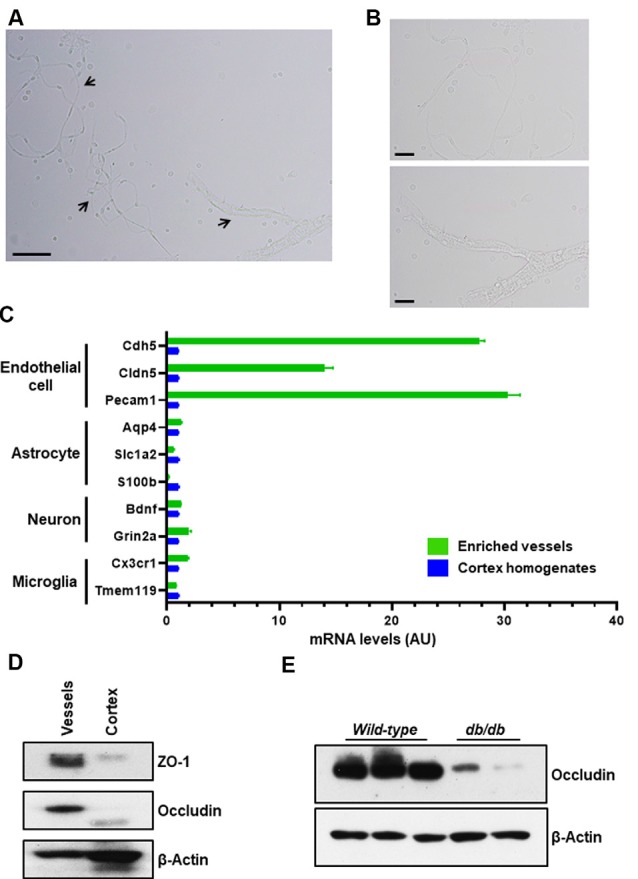
Purity of enriched brain microvessels. The enriched microvessels were examined by light microscope, bright-field directly after isolation and resuspension. **(A)** 100× magnification. Arrows indicate different sizes of microvessel fragments isolated. Scale bar, 50 μm. **(B)** 400× magnification. Top insert depicts a single stretched out capillary. The bottom insert depicts a capillary network attached to smooth muscle cells. Scale bar, 20 μm. **(C)** Endothelial cell markers (Cdh5, Cldn5, and Pecam1), astrocyte markers (Aqp4, Slc1a2 m, and S100b), neuron markers (Bdnf and Grin2a), and microglia markers (Cx3cr1 and Tmem119) were compared between the enriched brain microvessels and the cortex homogenates using qPCR quantification. Data are mean ± SEM, *n* = 2 mice per experiment from two independent experiments. **(D)** Representative Western blot shows that tight junction proteins, ZO-1 and Occludin, were enriched in isolated microvessel fragments compared to the cortex homogenates. The cytoskeletal β-actin was used as a control. Ten microgram of total protein preparation was examined. Data presented are from four independent experiments. **(E)** Representative Western blot of enriched brain microvessels isolated from wild-type C57BL/6 female mice (three samples) and *db*/*db* female mice (two samples) at age of 9 months. The tight junction protein, Occludin, was diminished in the *db*/*db* group. *n* = 2 mice per experiment from two independent experiments.

To isolate microvessels from the mouse cerebral cortex, as shown in Figure 1A, we adapted the protocol initially described by Miller, Bauer, and Hartz groups (Miller et al., 2000; Hartz et al., 2004, 2018b; Wang et al., 2010, 2014). The isolated microvessel fragments using mechanical homogenization described by Miller et al. (2000) retain P-glycoprotein, an ATP-dependent efflux transporter of the BBB and maintain the functional transport activity (Hartz et al., 2010, 2018b; Wang et al., 2010, 2014). We modified the Ficoll density-gradient and homogenization strokes to obtain optimal sizes of microvessel fragments from a pool of dissected cortices. The isolation protocol is overviewed in Figure 1B. The procedures described in Figure 2 yielded intact microvessel fragments as examined under a light microscope, although there was some brain parenchymal debris observed (Figures 3A,B). We validated the purity of isolated microvessels by examining the unique mRNAs expressed in different cell types. The isolated microvessels showed a 30-fold enrichment of endothelial cell-specific genes, *Pecam1*, *Cldn5*, and *Cdh5*, compared to other cell types, such as astrocytes, neurons, and microglia (Figure 3C). When compared to protein homogenates of the cerebral cortex, the isolated microvessels showed enrichment of tight junction protein, Zonula Occludens (ZO-1) and Occludin, indicating the intact barrier structure of microvessel fragments (Figure 3D). At this point, the isolated microvessel fragments can be used for molecular or biochemical assays to examine changes of tight junction proteins in a disease model. We examined a well-characterized model of human obesity and type 2 diabetes, which carries the mutated leptin receptor (*db*) alleles (Bahary et al., 1990). The *db*/*db* mouse has been reported exhibiting comorbid phenotypes of obesity, diabetes, and cognitive deficits (Erion et al., 2014; Stranahan et al., 2016; Cope et al., 2018). The cerebral vascular abnormalities and BBB damage were suggested contributing to the impairment of cognition observed (Niedowicz et al., 2014; Stranahan et al., 2016). We used this model as our validation studies for this isolation protocol. We isolated brain microvessels from *db*/*db* mice as procedures described above and examined the tight junction protein, Occludin (Figure 3E). The levels of Occludin in the microvessel fragments of *db*/*db* mice were significantly reduced compared to the controls. These results demonstrated the applications of this protocol to examine the vascular pathology in a dementia mouse model.

**Figure 4:**
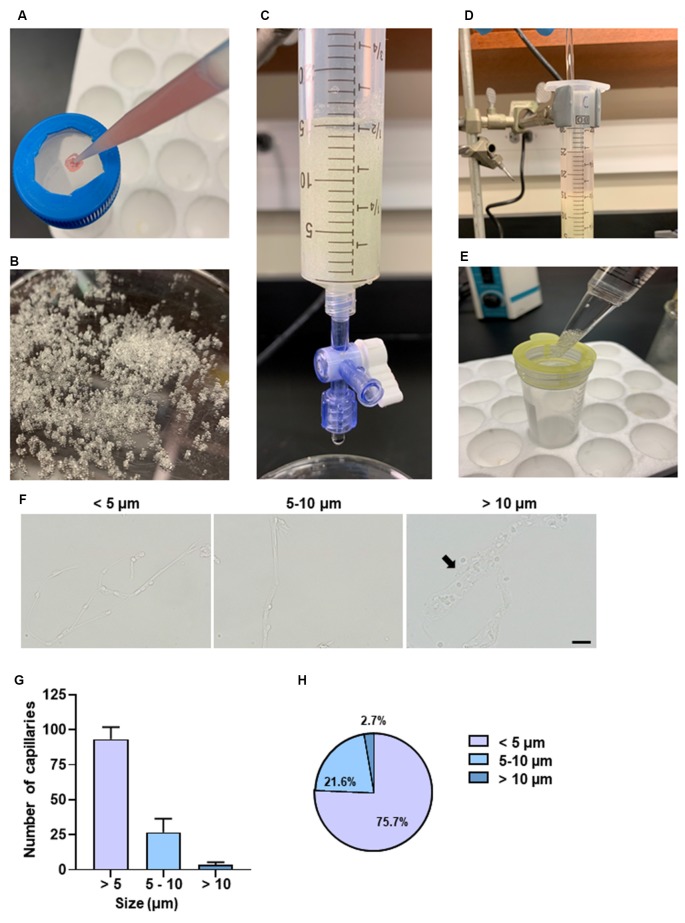
Purification of brain capillaries and characterization of capillary size. **(A)** Passing pellet resuspensions through a 300 μm nylon mesh. **(B)** Example of glass beads used. **(C)** Collection syringe with adjustable flow rate has 15 ml glass beads supported by a 30 μm nylon mesh. **(D)** Gentle agitation using a glass stirrer is necessary for microvessels to adhere to glass beads. **(E)** Filtering solution with a 100 μm filter to remove excess glass beads. **(F)** Representative bright-field images of different sizes of capillaries. The diameters of the capillaries were measured. A total of 70–120 capillaries was examined per experiment. Three independent experiments were performed. Arrow points to the representative capillary with a size between 10 and 20 μm. **(G)** The size of the capillaries was examined from each experimental preparation. Data are mean ± SEM *n* = 5 mice per experiment from three independent experiments. **(H)** Percentages of capillaries based on size. Data are mean ± SEM *n* = 5 mice per experiment from three independent experiments.

**Figure 5:**
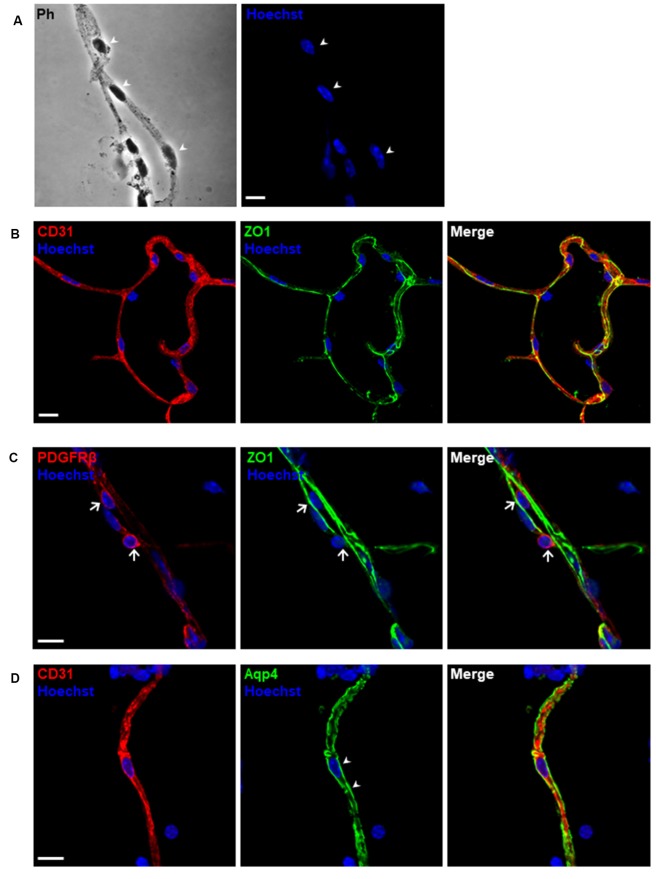
Morphological characterization of purified microvessels. Confocal images of immunostaining for different markers of blood-brain barrier (BBB) cell components. **(A)** Phase-contrast (Ph) image of microvessels. The cell nuclei shown by arrowheads are depicted as blue using Hoechst 33342 staining (1 μg/ml). **(B–D)** Isolated microvessels retain most of the *in situ* structure. **(B)** Endothelial markers, CD31 (red) and tight junction proteins, ZO-1 (green) were detected. **(C)** Pericyte markers, PDGFRβ (red) were detected adhering to the surface of an isolated capillary, as shown by arrows. **(D)** Astrocyte end-feet membranes, pointed by arrowheads, remain attached to the microvessel fragment. Scale bar, 10 μm. The data presented are from three independent experiments.

To exclude unwanted cellular debris, further purification of the enriched microvessel fragments was accomplished *via* the filtration through the glass beads column as shown in Figures 4A–E. We examined the diameters of the microvessel fragments using a bright-field light microscope. The majority of the microvessel fragments captured were less than 10 μm in diameter (Figures 4F,G). We observed that 75.7% of fragments were less than 5 μm, and 21.6% of fragments were between 5 and 10 μm (Figure 4H). The results suggested that most of the purified microvessel fragments are brain parenchymal capillaries. This filtration facilitated obtaining stretched and untangled capillaries that are suitable for imaging analyses (Figure 5A). We performed immunostaining to characterize the morphology of purified capillaries. Using the immunostaining of endothelial cell marker, CD31, we were able to visualize homogeneously labeled endothelial cells within the intact structure of capillaries (Figure 5B). Also, the tight junction protein, ZO-1, was co-labeled with CD31+ endothelial cells with no apparent discontinuity, suggesting that the structure of endothelial tight junctions were preserved in our capillary preparation. The surface of brain capillaries is covered by pericytes and astrocyte end-feet that seals the BBB entirely resulting in the unique characteristics of the cerebrovascular system (Daneman et al., 2010). We performed immunostaining of pericytes and astrocyte end-feet using makers, PDGFRβ and Aquaporin-4 (App4) respectively, to examine the sheathing of isolated capillaries. The PDGFRβ+ pericytes were shown adhered to the surface of isolated capillaries (Figure 5C). The water channel Aqp4 is concentrated at the astrocyte end-feet membrane, serving as an influx route of water to maintain homeostasis (Papadopoulos and Verkman, 2013; Camassa et al., 2015). We found that the Aqp4+ end-feet membrane outlined the wall of isolated capillaries (Figure 5D). Together, these results suggest that our optimized protocol using mechanical homogenization, but not enzymatic digestion, preserves the structure of the BBB that closely mimic *in vivo* situations.

**Figure 6:**
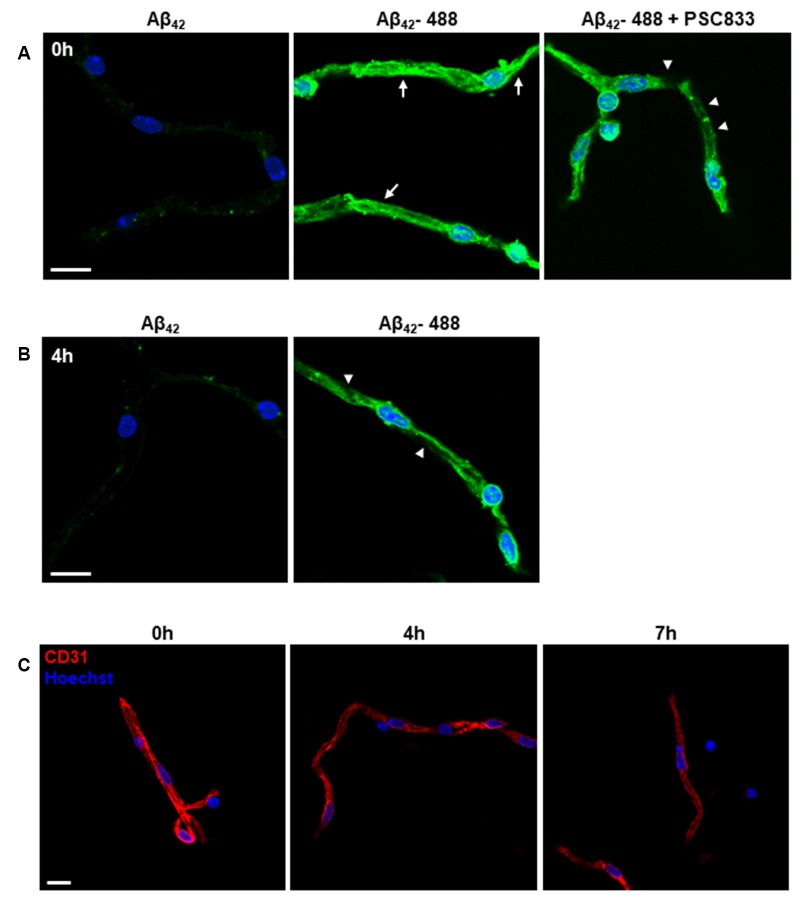
Functional transport characterization of purified microvessels. **(A,B)** Confocal images of Aβ_42_ transport in purified capillaries. **(A)** Freshly prepared capillaries were incubated with 5 μM HiLyte Fluor-488 Aβ_42_ (Aβ_42–488_) peptide or non-labeled Aβ_42_ peptide (as control) in modified PBS at room temperature for 1.5 h. After incubation, capillaries were washed and processed for imaging analysis. Capillary luminal fluorescence (green) of Aβ_42–488_ was examined using a confocal microscope. Cell nuclei are shown as blue using Hoechst 33342 staining (1 μg/ml). A P-glycoprotein inhibitor, PSC833, was co-incubated with Aβ_42–488_ to suppress the transport of Aβ_42–488_ mediated by the P-glycoprotein. Representative images from four independent experiments are shown. *n* = 5 mice per experiment. Arrows indicate the transport of Aβ_42–488_ into the capillary luminal space. Arrowheads indicate the reduced green fluorescence in the capillary luminal space. Scale bar, 10 μm. **(B)** Transport of Aβ_42–488_ in capillaries at 4 h after purification. The assay condition was the same as described in **(A)**. Arrowheads indicate decreased capillary luminal green fluorescence. **(C)** Endothelial markers, CD31, was used to examine the viable endothelial cells in purified capillaries at 0 h, 4 h, and 7 h after purification. The cell nuclei are shown as blue using Hoechst 33342 staining (1 μg/ml). Scale bar, 10 μm.

The purified capillaries using this protocol can be applied for functional transport assays to test small-molecule drugs (Wang et al., 2010, 2014). AD is an age-associated neurodegenerative disorder characterized by the buildup of amyloid-β (Aβ) plaques. The accumulation of Aβ as the result of unbalanced Aβ generation subsequent clearance is reported to contribute to the pathogenesis of AD (Bell et al., 2007; Mawuenyega et al., 2010; Zlokovic, 2011). We used the transport of human Aβ_42_ peptide to validate the function of the purified microvessel fragments (Figure 6A). We first used freshly isolated capillaries to examine the transport of Aβ_42_ (Hartz et al., 2010). We incubated HiLyte-Fluor 488 labeled Aβ_42_ peptide (Aβ_42–488_) or non-labeled Aβ_42_ peptide with purified capillaries in modified PBS at room temperature for 1.5 h. We observed the accumulated fluorescence of Aβ_42–488_ in the luminal space of capillaries, compared to non-labeled Aβ_42_ peptide. The P-glycoprotein inhibitor, PSC833, to suppress the transport of Aβ_42–488_ was shown. We tested the ability of purified capillaries to transport Aβ_42–488_ at 4h after purification (Figure 6B). The purified capillaries were incubated with modified PBS at room temperature for 4 h and then subjected to the transport assay for 1.5 h as described above. The reduction of Aβ_42–488_ fluorescence in the luminal space suggested that the purified capillaries have reduced function and viability at 5.5 h after purification, as suggested in previous studies (Bauer et al., 2008; Hartz et al., 2010; Hartz et al., 2018b). We also found that the CD31 immunostaining signal in purified capillaries is reduced at 4 h and almost diminished at 7 h after purification (Figure 6C). Together, these results suggested that the purified capillaries retain the transport function of BBB as *in vivo*, and the viability of purified capillaries can be preserved at modified PBS for up to 5 h.

Transcriptional profiling of the brain vascular endothelial cells provides new insights into the molecular mechanisms of vascular function in health and diseases at a whole-genome level (Guo et al., 2012; Zhang et al., 2014; Sabbagh et al., 2018). The fluorescence-activated cell sorting (FACS) technique enables to purify pre-defined cell populations based on the expression of surface markers or fluorescent reporters (Crouch and Doetsch, 2018; Jung et al., 2018; Munji et al., 2019). The selection of appropriate markers is often challenging, as some common markers being shared among BBB component cells and other types of brain parenchymal cells, such as oligodendrocyte progenitor cells. The enzymatic tissue dissociation before FACS purification is a critical step, as some epitopes required for immunostaining are degraded in a time-dependent manner during tissue dissociation (Crouch and Doetsch, 2018).

**Figure 7:**
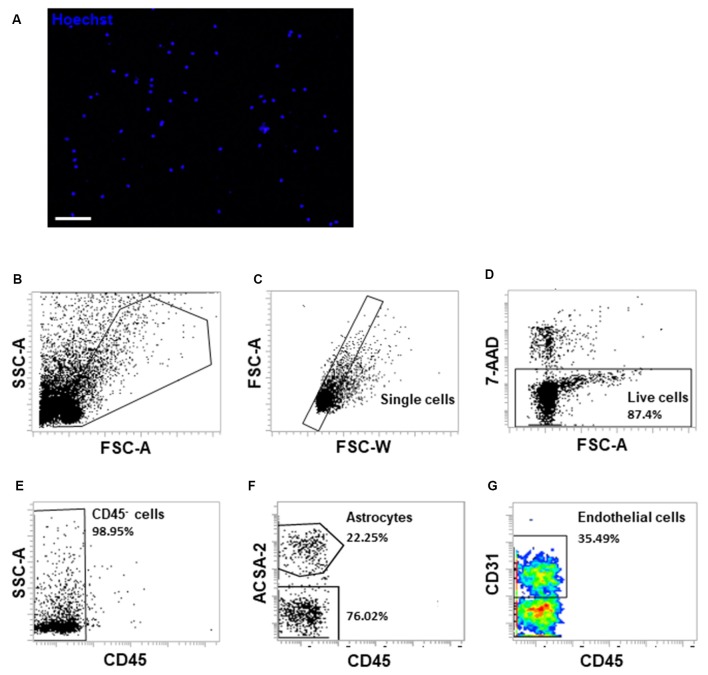
Fluorescence-activated cell sorting (FACS) analysis of single-cell suspensions of isolated brain microvessels. **(A)** Single-cell suspensions were examined under a fluorescent microscope immediately after resuspension. The cell nuclei were stained with Hoechst 33342 (1 μg/ml). Scale bar, 50 μm. **(B)** Single-cell suspensions were gated on FSC-A and SSC-A to exclude cell debris. **(C)** Cells were gated on FSC-W and FSC-A to exclude cell doublets or aggregates. **(D)** 7-AAD uptake indicates dead cells, which account for approximately 13% of cells. **(E)** CD45- cells were gated to exclude monocytes/macrophages and brain resident microglial cells. **(F,G)** ACSA-2 and CD31 staining were applied to examine different cell types within the single-cell suspensions generated from isolated microvessel fragments. Representative FACS images were from three to four independent experiments.

The development of single-cell technologies opens the opportunity to map cellular heterogeneity within BBB component cells and to unravel the molecular signatures of previously unrecognized cell populations or functional states in various subregions of the brain (Saliba et al., 2014; Cembrowski, 2019; Kalluri et al., 2019). Yousef and colleagues recently applied enzymatic digestion procedures following flow cytometry sorting to characterize the molecular phenotype of brain endothelial cells in the aging mouse brain (Czupalla et al., 2018; Yousef et al., 2018, 2019). Since we obtained the structurally and functionally intact brain capillaries, we further optimized the procedures to generate single-cell suspensions with relatively shortened time (15 vs. 50 min) and lowered dose of collagenase (0.5 mg/ml vs. 1 mg/ml) digestion step to minimize the potential of cell activation compared with other methods (Czupalla et al., 2018; Yousef et al., 2018; Kalluri et al., 2019). We used 10% fetal bovine serum to inactivate collagenase activity and stabilize cells rapidly during collection procedures. Following the procedures described, we were able to generate single-cell suspensions as examined under a fluorescence microscope (Figure 7A). The viable cells accounted for approximately 87% of the total single cells using 7-AAD uptake analysis (Figures 7B–D). We observed heterogeneous cell populations with minimal contamination of 2% CD45+ immune cell populations (Figure 7E). As we observed astrocyte end-feet covered the wall of isolated brain capillaries, we used astrocyte cell surface antigen-2 (ACSA-2) and CD31 to characterize BBB component cell types within the single-cell suspensions (Figures 7F,G). Cumulatively, this protocol is highly reproducible. We were able to yield an average of 4–6 × 10^5^ cells from a pool of cerebral cortices collected from four to five adult mice.

### Potential Pitfalls and Troubleshooting

This protocol describes the relative ease in isolating the microvessels from the adult mouse brain. Here, we list several conditions that will facilitate the preservation of cellular interactions between microvascular endothelial cells and mural cells, which is critical for maintaining the molecular phenotypes of BBB component cells.

- Keep all tools and buffers ice-cold to maintain a high viability (>80%). Also, performing the majority of the steps in a 4°C cold room will help keeping the cells viable.
- Each isolation of cerebral microvascular fragments should include experimental and control groups. The number of mice to use per group depends on downstream analyses. The protocol described above, especially the volume of Ficoll, is good for 3–5 mice per group. For biochemical analyses, such as RNA or protein quantifications, a pool of dissected cortices from two young mice (3–4-month-old male or female) is the amount required. For imaging of functional assays and single-cell analyses, a pool of dissected cortices from four to five young mice (3–4-month-old male or female) is the amount required.
- To maintain the high viability and functionality, the maximum number of groups that can be isolated at one time is two sets of mice (4–8 mice total). Increasing the total time for the procedure is detrimental to the procedure. We found that once the cells are in a single-cell suspension, the cells will begin loss of viability after 30 min and will affect the results.
- Modified PBS and the Ficoll must be made the day before the experiment and not any longer. Also, the sodium pyruvate and the glucose can only be added on the day of the experiment and must be used within the day of the experiment. Likewise, the 1% BSA solution is made right after the sodium pyruvate and the glucose are added to the modified PBS.
- The flow rate during the glass beads section is critical for the microvessel fragments to attach to the glass beads. If the flow rate is too fast, the capillaries will not adhere to the glass beads.
- The glass beads can be reused up to five times before brain parenchyma will contaminate the beads, preventing the capillary segments from adhering to the beads. Upon completion of the experiment, the glass beads can be washed by adding deionized water first followed by 95% ethanol to the glass beads, swirling first, then dumping the excess ethanol out. Rinse the glass beads twice with deionized water. Dry beads by immersing them in 100% ethanol overnight. The glass beads must be fully dry before being used again.

## Discussion

This protocol will be of significant interest in investigating molecular mechanisms that lead to BBB dysfunction. It describes in detail how to isolate brain microvessels while the isolated fragments retain the structural and functional integrity of the BBB. Freshly isolated microvessels can be used to characterize barrier dysfunction or capillary permeability if a disease mouse model is applied (Hartz et al., 2012). We validated the application of this protocol using a mouse model exhibiting BBB damage and cognitive impairment. Importantly, this protocol describes procedures to generate single-cell suspensions from isolated microvessels. The procedures require approximately 3–3.5 h in total depending on the experiment groups of mice used. The shorten time required from the described protocol can significantly improve the quality of RNA obtained. Moreover, the purified microvessel fragments were demonstrated retaining the *in vivo* structure and function of transport. The highly viable single-cell suspensions obtained from microvessels that retain *in situ* structure provide a valuable resource for identifying unique subpopulations of BBB component cells that might be associated with disease progression using single-cell analyses, such as RNA-sequencing (Yousef et al., 2019), DNA methylation (Hui et al., 2018), or ATAC-sequencing (Lalonde et al., 2019).

This protocol is optimized for the isolation of microvessel fragments and capillaries from the cerebral cortex of the adult mouse brain. However, it is crucial to consider that the dissection of the brain region of interest is required. If other brain sub-regions, such as sub-ventricular zone containing neurogenic niche or highly vascularized hippocampus, are of interest, the microdissection skill, fine forceps, and a dissection microscope will be required. The procedures described during brain harvest remove the thin slices from the dorsal surface of the brain, which contains the subdural meninges. Since we were unable to peel off the pia/arachnoid mater within the brain parenchyma, the isolated microvessels do not contain extra-parenchymal meningeal or large vessels on the surface of the brain as demonstrated in the size characterization of purified fragments. For the study interest of meningeal vessels, other methods should be followed (Park et al., 2011; Faraco et al., 2019).

## Conclusion

Developing a protocol to isolate microvessel fragments is vital to generate a model to study the BBB dysfunction in diseases. Since the capillaries make up for roughly 1% of the whole brain in humans, using crude brain homogenates will most likely generate weak signals to study the BBB dysfunction as shown in our results and reported literature (Bauer et al., 2008; Hartz et al., 2018a). Here, we show that enrichment of the cerebral capillaries *via* a density gradient and glass beads opens the door for future experiments to examine and characterize the neurovascular unit through various assays and molecular characterization, such as single-cell analysis.

## Data Availability Statement

All datasets generated for this study are included in the article.

## Ethics Statement

The animal study was reviewed and approved by Purdue Animal Care and Use Committee.

## Author Contributions

J-HY and I-CY conceived the study. HP, XW, and I-CY designed the experiments. HP, XW, P-CK, DF, and BS performed the experiments and analyzed the data. HP wrote the manuscript. J-HY, F-LC, and I-CY reviewed and edited the manuscript.

## Conflict of Interest

The authors declare that the research was conducted in the absence of any commercial or financial relationships that could be construed as a potential conflict of interest.
